# Age- and Diagnosis-Based Trends for Unplanned Pediatric Rehospitalizations in the United States

**DOI:** 10.7759/cureus.20181

**Published:** 2021-12-05

**Authors:** Nupur Amritphale, Amod Amritphale, Deepa Vasireddy, Mansi Batra, Mukul Sehgal, David Gremse

**Affiliations:** 1 Department of Pediatrics, University of South Alabama, Mobile, USA; 2 Medicine/Cardiovascular Disease, University of South Alabama, Mobile, USA; 3 Pediatrics, Pediatric Group of Acadiana, Lafayette, USA; 4 Critical Care Medicine, University of South Alabama, Mobile, USA

**Keywords:** depression, inflammatory bowel disease, diabetes mellitus, asthma, cancer, pediatric hospital medicine, pediatric rehospitalizations

## Abstract

Background and objectives: Hospital readmission rate helps to highlight the effectiveness of post-discharge care. There remains a paucity of plausible age-based categorization especially for ages below one year for hospital readmission rates.

Methods: Data from the 2017 Healthcare Cost and Utilization Project National Readmissions Database was analyzed for ages 0-18 years. Logistic regression analysis was performed to identify predictors for unplanned early readmissions.

Results: We identified 5,529,389 inpatient pediatric encounters which were further divided into age group cohorts. The overall rate of readmissions was identified at 3.2%. Beyond infancy, the readmission rate was found to be 6.7%. Across all age groups, the major predictors of unplanned readmission were cancers, diseases affecting transplant recipients and sickle cell patients. It was determined that reflux, milk protein allergy, hepatitis and inflammatory bowel diseases were significant diagnoses leading to readmission. Anxiety, depression and suicidal ideation depicted higher readmission rates in those older than 13 years. Across ages one to four years, dehydration, asthma and bronchiolitis were negative predictors of unplanned readmission.

Conclusions: Thirty-day unplanned readmissions remain a problem leading to billions of taxpayer dollars lost per annum. Effective strategies for mandatory outpatient follow-up may help the financial aspect of care while also enhancing the quality of care.

## Introduction

Readmission rates have long been used by clinicians, hospital systems, and health care commissions as a quality indicator. According to the Centers for Medicare and Medicaid Services (CMS), about one in five Medicare patients discharged from the hospital were readmitted within 30 days [1]. With adequate measures and practicing preventive care, it has been seen that approximately 75% of them are potentially preventable [2].

The readmission rates amongst adults have been vastly studied, however, the pediatric world has limited research to its name analyzing all causes [3,4]. Also, it has been suggested that pediatric data is becoming increasingly concentrated in large academic centers [5]. There is an increasing percentage of patients who are medically insured and there is also a better patient-centered medical home provision along with accountable and coordinated care at multiple academic facilities [6]. Due to the improvement in neonatal-perinatal healthcare strategies, a vast majority of children live longer with their chronic ailments and also develop long-term consequences of the disease. This also however leads to an increase in the rate of unplanned readmissions [7].

For this study, we used nationally representative data during the year 2017 to determine more recent trends in pediatric hospitalization and readmission. We have included all ages and stratified our study based on the age of patients admitted to determine age-specific causes for readmissions. This study is composed of only unplanned readmissions and includes patients rehospitalized within 30 days to give us a better picture of their etiology and therefore help us implement strategies in the future to limit readmissions.

This article was previously posted to the MedRxiv preprint server on November 14, 2021.

## Materials and methods

The National Readmission Database (NRD) is a collection of all-age, all-payer discharges represented at the national level from U.S. nonfederal hospitals produced by the Healthcare Cost and Utilization Project of the Agency for Healthcare Research and Quality [8]. This database is composed of discharge-level hospitalization data from 28 geographically dispersed states across the United States of America. Approximately, it has over 5 million inpatient pediatric encounters for the year 2017 (weighted database for national representation). The dataset used in the present study represents 60% of the U.S. population and 58.2% of all U.S. hospitalizations. Every year, hospitalizations and rehospitalizations can be determined, using a de-identified unique patient linkage number assigned to each patient, which enables tracking of patients across hospitals within a state. Individual patients in the NRD are assigned up to 40 diagnosis codes and 25 procedure codes for each hospitalization. The study was approved for exempt status by the Institutional Review Board at the University of South Alabama.

ICD-10 procedure & diagnosis codes (International Classification of Diseases, hereafter termed ICD-10 codes) and Clinical Classification Software Refined (CCSR) codes were used to identify the pertinent discharge level diagnosis, procedures and organ system information. The data was divided into age groups 0 to one year, one to four years, five to 12 years and 13 to 18 years respectively for analysis purposes. The information for the most common causes of admission for respective age groups was obtained from the Healthcare Cost and Utilization Project. Patients who died during their initial hospitalizations as well as those who were planned elective readmissions within 30 days of prior discharge were excluded. The cohort patients admitted in the month of December for the index admission were also excluded, as they may not have 30 days of follow-up, leading to immortal time bias.

ICD-10 codes and CCSR codes were used to define organ system disorders and various comorbidities. The All Patient Refined-Diagnosis Related Group (APR DRG) Mortality risk score and APR DRG Severity of Illness calculations were also performed in the analysis. The costs were determined by multiplying the hospital charges with the Agency for Healthcare Research and Quality’s all-payer cost-to-charge ratios for each hospital. All ICD-10 and CCSR codes used in this study shall be made available upon request. The causes of readmission were determined by the first diagnosis on the basis of CCSR codes.

Statistical analysis was performed using IBM SPSS Statistics for Windows, version 1.0.0.1327 (IBM Corp., Armonk, NY, USA) using 2-sided tests and a significance level of 0.05. Within the time periods, baseline characteristics of participants were examined and tested for statistical differences using the Pearson Chi Square test for categorical variables and Mann-Whitney U-Test for continuous variables with no readmission as the reference group. Logistic regressions were used to evaluate the predictors of readmission within respective age groups. The odds ratio was calculated by using logistic regression. We followed the methodology as has been previously described in well-written studies [4,9-14].

## Results

Population characteristics and descriptive results

Our nationally registered database had a sample size of a total 5,529,389 inpatient encounters from January through December 2017 consisting of all ages from birth up to 18 years of age. The patients who died during their index admission as well as those who were discharged after November 30th were excluded from the index hospitalizations to remain within the threshold of 30-day readmissions. The remaining sample included a total of 5,096,320 patients who were discharged home alive. The total readmission rate for all age groups, all causes, was 3.2%. Readmission rates among infants were 2.1% and tripled to 6.7% beyond infancy.

Cost analysis and length of stay results

For ages below one, the median hospital cost of index admission encounters was $1,363 [interquartile range (IQR) $894 - $2,370] while for readmissions it was $11,834 [IQR $5,738 - $23,471]. The median cost of index hospitalization for those with 30-day readmission was $1,734 [IQR $1,734 - $4,961] compared with $1,356 [IQR $892 - $2,343] for those with no readmission [p<0.001]. The total charges for the index encounters and 30-day unplanned readmission encounters amount to over $71 billion and $1.8 billion respectively. Median length of stay for index admission with and without 30-day readmission was two days [IQR two to three days] and two days [IQR one to four days] respectively. There was significant variation in cost and length of stay among different age groups (Table 1, Table 2).

**Table 1:** Baseline characteristics, demographics, organ system involvement and most common diagnosis for index pediatric admissions in the United States for age groups <1 and 1-4 years. a: Per HCUP guidance, cell clusters with encounter numbers less than 10 were not reported Abbreviations: CKD: Chronic Kidney Disease; AKI: Acute Kidney Injury; IBD: Inflammatory Bowel Disease; HIV: Human Immunodeficiency Virus; UTI: Urinary Tract Infection.

	Age <1					Age 1-4				
	No early readmissions (n= 3705889 ; 97.9%)	30 day readmission (n= 75778 ; 2.1%)	Overall (n= 3781667)	P value	Odds ratio (95% CI)	No early readmissions (n= 264667 ; 90.7%)	30 day readmission (n= 21230 ; 9.3%)	Overall (n= 285897)	P value	Odds ratio (95% CI)
Age (yrs) (median [IQR])	0 (0-0)	0 (0-0)	0 (0-0)	-		2 (1-3)	2 (1-3)	2 (1-3)	-	
Gender										
Female	1791312 (48.3%)	32674 (43.1%)	1823986 (48.2%)	<0.001	0.810 (0.799 - 0.822)	114909 (43.4%)	9203 (43.3%)	124112 (43.4%)	0.849	0.997 (0.970 - 1.026)
Male	1914577 (51.7%)	43104 (56.9%)	1957681 (51.8%)	(reference)	(reference)	149758 (56.6%)	21027 (56.7%)	161785 (56.6%)	(reference)	(reference)
Elective	56542 (1.5%)	2615 (3.5%)	59157 (1.6%)	<0.001	2.308 (2.218 - 2.402)	46160 (17.5%)	4283 (20.4%)	50443 (17.7%)	<0.001	1.203 (1.162 - 1.246)
Weekend admission	783985 (21.2%)	17074 (22.5%)	801059 (21.2%)	<0.001	1.084 (1.065 - 1.103)	59725 (22.6%)	4167 (19.6%)	63892 (22.3%)	<0.001	0.838 (0.809 - 0.868)
Primary expected payer				<0.001					<0.001	
Medicare	15311 (0.4%)	208 (0.3%)	15519 (0.4%)			898 (0.3%)	156 (0.7%)	1054 (0.4%)		
Medicaid	1759706 (47.5%)	40726 (53.8%)	1800432 (47.7%)			162122 (61.4%)	13033 (61.4%)	175155 (61.4%)		
Private	1688188 (45.6%)	30860 (40.8%)	1719048 (45.5%)			88449 (33.5%)	7226 (34.1%)	95675 (33.5%)		
Self-pay	136226 (3.7%)	2002 (2.6%)	138228 (3.7%)			5415 (2%)	295 (1.4%)	5710 (2%)		
No charge	2446 (0.1%)	27 (0%)	2473 (0.1%)			140 (0.1%)	11 (0.1%)	151 (0.1%)		
Other	100662 (2.7%)	1890 (2.5%)	102552 (2.7%)			7193 (2.7%)	491 (2.3%)	7684 (2.7%)		
Quartile of median household income				<0.001					<0.001	
0–25^th^	881812 (24%)	17716 (23.5%)	899528 (23.9%)			86453 (33%)	6663 (31.6%)	93116 (32.9%)		
26^th^ –50^th^	974100 (26.5%)	20239 (26.9%)	994339 (26.5%)			73563 (28%)	6292 (29.9%)	79855 (28.2%)		
51^st^–75^th^	1032625 (28.1%)	21681 (28.8%)	1054306 (28.1%)			61114 (23.3%)	5215 (24.8%)	66329 (23.4%)		
76^th^ –100^th^	792419 (21.5%)	15667 (20.8%)	808086 (21.5%)			41201 (15.7%)	2894 (13.7%)	44095 (15.6%)		
Comorbidities										
Dehydration	53026 (1.4%)	2660 (3.5%)	55686 (1.5%)	<0.001	2.506 (2.409 - 2.607)	50014 (18.9%)	2873 (13.5%)	52887 (18.5%)	<0.001	0.672 (0.645 - 0.699)
Cancer	30864 (0.8%)	1697 (2.2%)	32561 (0.9%)	<0.001	2.728 (2.596 - 2.866)	19304 (7.3%)	7006 (33%)	26310 (9.2%)	<0.001	6.261 (6.062 - 6.465)
Injury poisoning	3806 (0.1%)	50 (0.1%)	3856 (0.1%)	0.002	0.642 (0.486 - 0.849)	2517 (1%)	50 (0.2%)	2567 (0.9%)	<0.001	0.246 (0.186 - 0.325)
Transplant	310 (0%)	50 (0.1%)	360 (0%)	<0.001	7.892 (5.854 - 10.64)1	3242 (1.2%)	923 (4.3%)	4165 (1.5%)	<0.001	3.665 (3.402 - 3.949)
UTI	15019 (0.4%)	821 (1.1%)	15840 (0.4%)	<0.001	2.692 (2.508 - 2.889)	8657 (3.3%)	787 (3.7%)	9444 (3.3%)	0.001	1.138 (1.057 - 1.226)
CKD/AKI/Nephritis	6208 (0.2%)	791 (1%)	6999 (0.2%)	<0.001	6.286 (5.836 - 6.772)	5523 (2.1%)	949 (4.5%)	6472 (2.3%)	<0.001	2.196 (2.046 - 2.356)
Sickle cell	4646 (0.1%)	428 (0.6%)	5074 (0.1%)	<0.001	4.525 (4.097 - 4.997)	5879 (2.2%)	526 (2.5%)	6405 (2.2%)	0.015	1.118 (1.022 - 1.224)
Cardiomyopathy/dysrrhythmias	11142 (0.3%)	769 (1%)	11911 (0.3%)	<0.001	3.400 (3.159 - 3.659)	3137 (1.2%)	500 (2.4%)	3637 (1.3%)	<0.001	2.011 (1.828 - 2.212)
Intestinal infection	8794 (0.2%)	606 (0.8%)	9400 (0.2%)	<0.001	3.389 (3.120 - 3.681)	14024 (5.3%)	1122 (5.3%)	15146 (5.3%)	0.931	0.997 (0.937 - 1.062)
Appendiceal conditions	-^a^	-^a^	-^a^	-^a^	-^a^	2685 (1%)	212 (1%)	2897 (1%)	0.824	0.984 (0.855 - 1.133)
IBD	-^a^	-^a^	-^a^	-^a^	-^a^	258 (0.1%)	23 (0.1%)	281 (0.1%)	0.627	1.111 (0.725 - 1.703)
Hepatitis	-^a^	-^a^	-^a^	-^a^		100 (0%)	28 (0.1%)	128 (0%)	<0.001	3.494 (2.297 - 5.314)
GI other (digestive hepatobiliary diseases)	95814 (2.6%)	7538 (9.9%)	103352 (2.7%)	<0.001	4.162 (4.061 - 4.266)	59392 (22.4%)	8116 (38.2%)	67508 (23.6%)	<0.001	2.139 (2.077 - 2.202)
Epilepsy	13132 (0.4%)	1581 (2.1%)	14713 (0.4%)	<0.001	5.992 (5.684 - 6.316)	30306 (11.5%)	3630 (17.1%)	33936 (11.9%)	<0.001	1.595 (1.536 - 1.656)
Diabetes	-^a^	-^a^	-^a^	-^a^	-^a^	2224 (0.8%)	98 (0.5%)	2322 (0.8%)	<0.001	0.547 (0.447 - 0.670)
Endocrine other than diabetes	92353 (2.5%)	6936 (9.2%)	99289 (2.6%)	<0.001	3.942 (3.843 - 4.044)	78624 (29.7%)	7647 (36%)	86271 (30.2%)	<0.001	1.332 (1.294 - 1.372)
Cystic fibrosis	-^a^	-^a^	-^a^	-^a^	-^a^	999 (0.4%)	51 (0.2%)	1050 (0.4%)	0.001	0.636 (0.480 - 0.842)
Skin Subcutaneous	53491 (1.4%)	2589 (3.4%)	56080 (1.5%)	<0.001	2.415 (2.320 - 2.514)	36834 (13.9%)	2638 (12.4%)	39472 (13.8%)	<0.001	0.878 (0.841 - 0.915)
Psychiatric disorders	1090 (0%)	96 (0.1%)	1186 (0%)	<0.001	4.311 (3.499 - 5.312)	2335 (0.9%)	288 (1.4%)	2623 (0.9%)	<0.001	1.545 (1.366 - 1.748)
Congenital anomalies	555947 (15%)	18516 (24.4%)	574463 (15.2%)	<0.001	1.832 (1.802 - 1.863)	49195 (18.6%)	5925 (27.9%)	55120 (19.3%)	<0.001	1.696 (1.643 - 1.750)
Musculoskeletal	7760 (0.2%)	293 (0.4%)	8053 (0.2%)	<0.001	1.850 (1.646 - 2.079)	9046 (3.4%)	444 (2.1%)	9490 (3.3%)	<0.001	0.604 (0.548 - 0.665)
Asthma, bronchiolitis	84371 (2.3%)	4421 (5.8%)	88792 (2.3%)	<0.001	2.659 (2.578 - 2.743)	80663 (30.5%)	4117 (19.4%)	84780 (29.7%)	<0.001	0.549 (0.530 - 0.568)
Pneumonia	23748 (0.6%)	1535 (2%)	25283 (0.7%)	<0.001	3.206 (3.043 - 3.377)	36440 (13.8%)	1919 (9%)	38359 (13.4%)	<0.001	0.622 (0.593 - 0.653)
Respiratory failure/arrest	31998 (0.9%)	3051 (4%)	35049 (0.9%)	<0.001	4.817 (4.638 - 5.003)	24218 (9.2%)	2574 (12.1%)	26792 (9.4%)	<0.001	1.370 (1.312 - 1.430)
Hemolytic perinatal jaundice	824107 (22.2%)	19913 (26.3%)	844020 (22.3%)	<0.001	1.246 (1.226 - 1.267)	-^a^	-^a^	-^a^	-^a^	-^a^
Septicemia	7841 (0.2%)	606 (0.8%)	8447 (0.2%)	<0.001	3.802 (3.499 - 4.131)	4938 (1.9%)	689 (3.2%)	5627 (2%)	<0.001	1.764 (1.627 - 1.913)
Neutropenia	9156 (0.2%)	633 (0.8%)	9789 (0.3%)	<0.001	3.401 (3.137 - 3.688)	5140 (1.9%)	1317 (6.2%)	6457 (2.3%)	<0.001	3.339 (3.138 - 3.554)
APR DRG Mortality risk				<0.001					<0.001	
Minor likelihood of dying	3549349 (95.8%)	65265 (86.1%)	3614614 (95.6%)			204279 (77.2%)	10471 (49.3%)	214750 (75.1%)		
Moderate likelihood of dying	97623 (2.6%)	5510 (7.3%)	103133 (2.7%)			43188 (16.3%)	7564 (35.6%)	50752 (17.8%)		
Major likelihood of dying	42693 (1.2%)	3704 (4.9%)	46397 (1.2%)			12792 (4.8%)	2631 (12.4%)	15423 (5.4%)		
Extreme likelihood of dying	11679 (0.3%)	1197 (1.6%)	12876 (0.3%)			4000 (1.5%)	552 (2.6%)	4552 (1.6%)		
APR DRG Severity of Illness				<0.001					<0.001	
Minor loss of function (includes cases with no comorbidity or complications)	2598278 (70.1%)	42107 (55.6%)	2640385 (69.8%)			113472 (42.9%)	3470 (16.3%)	116942 (40.9%)		
Moderate loss of function	759309 (20.5%)	18117 (23.9%)	777426 (20.6%)			90551 (34.2%)	7388 (34.8%)	97939 (34.3%)		
Major loss of function	297355 (8%)	10736 (14.2%)	308091 (8.1%)			47172 (17.8%)	7662 (36.1%)	54834 (19.2%)		
Extreme loss of function	46402 (1.3%)	4717 (6.2%)	51119 (1.4%)			13064 (4.9%)	2699 (12.7%)	15763 (5.5%)		
Hospital bed size				<0.001					<0.001	
Small	595474 (16.1%)	11315 (14.9%)	606789 (16%)			35944 (13.6%)	3486 (16.4%)	39430 (13.8%)		
Medium	1062727 (28.7%)	17749 (23.4%)	1080476 (28.6%)			46616 (17.6%)	2725 (12.8%)	49341 (17.3%)		
Large	2047687 (55.3%)	46714 (61.6%)	2094401 (55.4%)			182106 (68.8%)	15019 (70.7%)	197125 (68.9%)		
Control/Ownership of Hospital				<0.001					<0.001	
Government, nonfederal	312803 (8.4%)	8538 (11.3%)	321341 (8.5%)			31972 (12.1%)	2488 (11.7%)	34460 (12.1%)		
Private, not-profit	3123182 (84.3%)	63317 (83.6%)	3186499 (84.3%)			217142 (82%)	18190 (85.7%)	235332 (82.3%)		
Private, invest-own	269904 (7.3%)	3923 (5.2%)	273827 (7.2%)			15553 (5.9%)	552 (2.6%)	16105 (5.6%)		
Hospital Urban Rural Designation				<0.001					<0.001	
Large metropolitan areas with at least 1 million residents	1971291 (53.2%)	43541 (57.5%)	2014832 (53.3%)			166418 (62.9%)	14692 (69.2%)	181110 (63.3%)		
Small metropolitan areas with less than 1 million residents	1407799 (38%)	28025 (37%)	1435824 (38%)			86772 (32.8%)	6236 (29.4%)	93008 (32.5%)		
Micropolitan areas	255480 (6.9%)	3385 (4.5%)	258865 (6.8%)			8322 (3.1%)	235 (1.1%)	8557 (3%)		
Not metropolitan or micropolitan (non-urban residual)	71319 (1.9%)	826 (1.1%)	72145 (1.9%)			3155 (1.2%)	66 (0.3%)	3221 (1.1%)		
Hospital teaching status				<0.001					<0.001	
Metropolitan non-teaching hospital	756340 (20.4%)	14144 (18.7%)	770484 (20.4%)			19211 (7.3%)	920 (4.3%)	20131 (7%)		
Metropolitan teaching hospital	2622750 (70.8%)	57422 (75.8%)	2680172 (70.9%)			233979 (88.4%)	20009 (94.2%)	253988 (88.8%)		
Non-metropolitan hospital	326799 (8.8%)	4212 (5.6%)	331011 (8.8%)			11476 (4.3%)	302 (1.4%)	11778 (4.1%)		
Discharge destination				<0.001					<0.001	
Home/self-care	3624806 (97.8%)	71475 (94.3%)	3696281 (97.7%)			251799 (95.1%)	18607 (87.7%)	270406 (94.6%)		
Home health care	27262 (0.7%)	1464 (1.9%)	28726 (0.8%)			2293 (0.9%)	382 (1.8%)	2675 (0.9%)		
Discharge against medical advice	887 (0%)	29 (0%)	916 (0%)			169 (0.1%)	15 (0.1%)	184 (0.1%)		
Length of stay and cost analysis										
Index admission length of stay (days) (median [IQR])	2(2-3)	2(2-3)	2(2-3)			2(1-4)	3(2-6)	2(1-4)		
Index admission hospital charge (U.S.$) (median [IQR])	4132(2724-7531)	5419(3269-16106)	4152(2732-7626)			16194(8826-32798)	27867(14161-60735)	16814(9062-34493)		
Index admission cost (U.S.$) (median [IQR])	1356(892-2343)	1734(1734-4961)	1363(894-2370)			4661(2626-9558)	8373(4200-18828)	4844(2691-10074)		
Re-admission length of stay (days) (median [IQR])			2 [1 – 4]					2 [2 – 5]		
Re-admission hospital charge (U.S.$) (median [IQR])			11834 [5738 – 23471]					25072 [13494 – 49233]		
Re-admission cost (U.S.$) (median [IQR])			3820 [1934 – 7717]					7583 [4019 – 14754]		

**Table 2:** Baseline characteristics, demographics, organ system involvement and most common diagnosis for index pediatric admissions in the United States for age groups 5-12 years and 13-18 years. a: Per HCUP guidance, cell clusters with encounter numbers less than 10 were not reported Abbreviations: CKD: Chronic Kidney Disease; AKI: Acute Kidney Injury; IBD: Inflammatory Bowel Disease; HIV: Human Immunodeficiency Virus; UTI: Urinary Tract Infection.

	Age 5-12					Age 13-18				
	No early readmissions (n= 348671 4; 91.1%)	30 day readmission (n= 26866 ; 8.9%)	Overall (n= 375537)	P value	Odds ratio (95% CI)	No early readmissions (n= 612189 ; 92.4%)	30 day readmission (n= 41030 ; 7.6%)	Overall (n= 653219)	P value	Odds ratio (95% CI)
Age(yrs) (median[IQR])	9 (7 -11)	9 (7 -11)	9 (7 -11)	-		16 (15-17)	16 (14-17)	16 (15-17)	-	
Gender										
Female	153367 (44%)	12301 (45.8%)	165668 (44.1%)	<0.001	1.075 (1.049 - 1.103)	382291 (62.4%)	23252 (56.7%)	405543 (62.1%)	<0.001	0.787 (0.771 - 0.803)
Male	195304 (56%)	14565 (54.2%)	209869 (55.9%)	(Reference)	(Reference)	229898 (37.6%)	17778 (43.3%)	247676 (37.9%)	(Reference)	(Reference)
Elective	69219 (19.9%)	4857 (18.2%)	74076 (19.8%)	0.803	0.893 (0.865 - 0.922)	138221 (22.7%)	6420 (15.7%)	144641 (22.2%)	<0.001	0.637 (0.620 - 0.654)
Weekend admission	69686 (20%)	4684 (17.4%)	74370 (19.8%)	<0.001	0.845 (0.818 - 0.873)	123435 (20.2%)	7659 (18.7%)	131094 (20.1%)	<0.001	0.909 (0.886 - 0.932)
Primary expected payer				<0.001					<0.001	
Medicare	1273 (0.4%)	206 (0.8%)	1479 (0.4%)			2399 (0.4%)	222 (0.5%)	2621 (0.4%)		
Medicaid	202808 (58.3%)	15427 (57.5%)	218235 (58.2%)			335184 (54.8%)	22766 (55.6%)	357950 (54.9%)		
Private	127088 (36.5%)	9925 (37%)	137013 (36.5%)			238669 (39%)	15885 (38.8%)	254554 (39%)		
Self-pay	6237 (1.8%)	462 (1.7%)	6699 (1.8%)			14951 (2.4%)	787 (1.9%)	15738 (2.4%)		
No charge	350 (0.1%)	22 (0.1%)	372 (0.1%)			654 (0.1%)	36 (0.1%)	690 (0.1%)		
Other	10354 (3%)	806 (3%)	11160 (3%)			19428 (3.2%)	1281 (3.1%)	20709 (3.2%)		
Quartile of median household income				0.044					0.028	
0–25^th^	111654 (32.3%)	8598 (32.4%)	120252 (32.3%)			196596 (32.5%)	12875 (31.8%)	209471 (32.4%)		
26^th^ –50^th^	97691 (28.3%)	7414 (27.9%)	105105 (28.3%)			172267 (28.5%)	11611 (28.7%)	183878 (28.5%)		
51^st^–75^th^	79410 (23%)	6034 (22.7%)	85444 (23%)			137877 (22.8%)	9352 (23.1%)	147229 (22.8%)		
76^th^ –100^th^	56504 (16.4%)	4514 (17%)	61018 (16.4%)			98406 (16.3%)	6681 (16.5%)	105087 (16.3%)		
Comorbidities										
Dehydration	35228 (10.1%)	2702 (10.1%)	37930 (10.1%)	0.809	0.995 (0.955 - 1.037)	33737 (5.5%)	2976 (7.3%)	36713 (5.6%)	<0.001	1.341 (1.290 - 1.394)
Cancer	26526 (7.6%)	8368 (31.1%)	34894 (9.3%)	<0.001	5.494 (5.338 - 5.654)	25830 (4.2%)	7642 (18.6%)	33472 (5.1%)	<0.001	5.196 (5.053 - 5.342)
Injury poisoning	4407 (1.3%)	59 (0.2%)	4466 (1.2%)	<0.001	0.172 (0.133 - 0.222)	13495 (2.2%)	348 (0.8%)	13843 (2.1%)	<0.001	0.380 (0.341 - 0.422)
Transplant	4807 (1.4%)	1212 (4.5%)	6019 (1.6%)	<0.001	3.379 (3.169 - 3.604)	3678 (0.6%)	728 (1.8%)	4406 (0.7%)	<0.001	2.989 (2.758 - 3.238)
UTI	11847 (3.4%)	1164 (4.3%)	13011 (3.5%)	<0.001	1.288 (1.211 - 1.369)	22345 (3.7%)	1648 (4%)	23993 (3.7%)	<0.001	1.105 (1.050 - 1.162)
CKD/AKI/Nephritis	10610 (3%)	1699 (6.3%)	12309 (3.3%)	<0.001	2.151 (2.040 - 2.268)	17774 (2.9%)	2314 (5.6%)	20088 (3.1%)	<0.001	1.999 (1.912 - 2.090)
Sickle cell	11600 (3.3%)	1403 (5.2%)	13003 (3.5%)	<0.001	1.601 (1.513 - 1.695)	13186 (2.2%)	2806 (6.8%)	15992 (2.4%)	<0.001	3.335 (3.198 - 3.478)
Cardiomyopathy/dysrrhythmias	4139 (1.2%)	483 (1.8%)	4622 (1.2%)	<0.001	1.524 (1.386 - 1.676)	8791 (1.4%)	954 (2.3%)	9745 (1.5%)	<0.001	1.634 (1.527 - 1.748)
Intestinal infection	9904 (2.8%)	1083 (4%)	10987 (2.9%)	<0.001	1.437 (1.348 - 1.532)	7372 (1.2%)	833 (2%)	8205 (1.3%)	<0.001	1.700 (1.581 - 1.828)
Appendiceal conditions	21425 (6.1%)	1102 (4.1%)	22527 (6%)	<0.001	0.653 (0.614 - 0.695)	18819 (3.1%)	976 (2.4%)	19795 (3%)	<0.001	0.768 (0.720 - 0.820)
IBD	2960 (0.8%)	444 (1.7%)	3404 (0.9%)	<0.001	1.963 (1.775 - 2.170)	9260 (1.5%)	1233 (3%)	10493 (1.6%)	<0.001	2.017 (1.899 - 2.143)
Hepatitis	346 (0.1%)	62 (0.2%)	408 (0.1%)	<0.001	2.329 (1.777 - 3.052)	759 (0.1%)	150 (0.4%)	909 (0.1%)	<0.001	2.956 (2.480 - 3.523)
GI other (digestive hepatobiliary diseases)	82100 (23.5%)	9290 (34.6%)	91390 (24.3%)	<0.001	1.716 (1.672 - 1.762)	104328 (17%)	11646 (28.4%)	115974 (17.8%)	<0.001	1.929 (1.887 - 1.973)
HIV	-^a^	-^a^	-^a^	-^a^	-^a^	320 (0.1%)	30 (0.1%)	350 (0.1%)	0.077	1.399 (0.962 - 2.034)
Epilepsy	40662 (11.7%)	3860 (14.4%)	44522 (11.9%)	<0.001	1.271 (1.226 - 1.317)	33686 (5.5%)	3196 (7.8%)	36882 (5.6%)	<0.001	1.451 (1.397 - 1.507)
Diabetes	14263 (4.1%)	703 (2.6%)	14966 (4%)	<0.001	0.630 (0.583 - 0.680)	30487 (5%)	2953 (7.2%)	33440 (5.1%)	<0.001	1.480 (1.423 - 1.539)
Endocrine other than diabetes	86410 (24.8%)	9382 (34.9%)	95792 (25.5%)	<0.001	1.629 (1.586 - 1.672)	139178 (22.7%)	13342 (32.5%)	152520 (23.3%)	<0.001	1.638 (1.603 - 1.673)
Cystic fibrosis	3240 (0.9%)	135 (0.5%)	3375 (0.9%)	<0.001	0.538 (0.453 - 0.640)	5275 (0.9%)	378 (0.9%)	5653 (0.9%)	0.207	1.070 (0.963 - 1.188)
Skin Subcutaneous	41746 (12%)	3236 (12%)	44982 (12%)	0.726	1.007 (0.969 - 1.046)	48431 (7.9%)	4063 (9.9%)	52494 (8%)	<0.001	1.279 (1.237 - 1.323)
Psychiatric disorders	54616 (15.7%)	5474 (20.4%)	60090 (16%)	<0.001	1.378 (1.336 - 1.421)	230563 (37.7%)	16455 (40.1%)	247018 (37.8%)	<0.001	1.108 (1.086 - 1.131)
Congenital anomalies	45860 (13.2%)	3907 (14.5%)	49767 (13.3%)	<0.001	1.124 (1.085 - 1.164)	34498 (5.6%)	3049 (7.4%)	37547 (5.7%)	<0.001	1.344 (1.294 - 1.397)
Musculoskeletal	24243 (7%)	1176 (4.4%)	25419 (6.8%)	<0.001	0.613 (0.577 - 0.650)	44322 (7.2%)	2123 (5.2%)	46445 (7.1%)	<0.001	0.699 (0.669 - 0.731)
Complications during childbirth	-^a^	-^a^	-^a^	-^a^	-^a^	68640 (11.2%)	1004 (2.4%)	69644 (10.7%)	<0.001	0.199 (0.186 - 0.212)
Asthma, bronchiolitis	87869 (25.2%)	5082 (18.9%)	92951 (24.8%)	<0.001	0.692 (0.671 - 0.715)	85458 (14%)	6496 (15.8%)	91954 (14.1%)	<0.001	1.159 (1.128 - 1.192)
Pneumonia	27009 (7.7%)	1507 (5.6%)	28516 (7.6%)	<0.001	0.708 (0.671 - 0.747)	14553 (2.4%)	1295 (3.2%)	15848 (2.4%)	<0.001	1.338 (1.263 - 1.418)
Respiratory failure/arrest	17156 (4.9%)	1600 (6%)	18756 (5%)	<0.001	1.224 (1.161 - 1.290)	14759 (2.4%)	1354 (3.3%)	16113 (2.5%)	<0.001	1.381 (1.305 - 1.462)
Septicemia	6226 (1.8%)	786 (2.9%)	7012 (1.9%)	<0.001	1.658 (1.537 - 1.787)	11051 (1.8%)	1060 (2.6%)	12111 (1.9%)	<0.001	1.443 (1.353 - 1.538)
Neutropenia	5554 (1.6%)	1305 (4.9%)	6859 (1.8%)	<0.001	3.154 (2.965 - 3.354)	3710 (0.6%)	870 (2.1%)	4580 (0.7%)	<0.001	3.553 (3.298 - 3.828)
APR DRG Mortality risk				<0.001					<0.001	
Minor likelihood of dying	279321 (80.1%)	14730 (54.8%)	294051 (78.3%)			528181 (86.3%)	28494 (69.4%)	556675 (85.2%)		
Moderate likelihood of dying	50289 (14.4%)	9161 (34.1%)	59450 (15.8%)			56327 (9.2%)	9145 (22.3%)	65472 (10%)		
Major likelihood of dying	13777 (4%)	2308 (8.6%)	16085 (4.3%)			17182 (2.8%)	2513 (6.1%)	19695 (3%)		
Extreme likelihood of dying	4147 (1.2%)	548 (2%)	4695 (1.3%)			6246 (1%)	639 (1.6%)	6885 (1.1%)		
APR DRG Severity of Illness				<0.001					<0.001	
Minor loss of function (includes cases with no comorbidity or complications)	140031 (40.2%)	5013 (18.7%)	145044 (38.6%)			239977 (39.2%)	9439 (23%)	249416 (38.2%)		
Moderate loss of function	131239 (37.6%)	10844 (40.4%)	142083 (37.8%)			267393 (43.7%)	19420 (47.3%)	286813 (43.9%)		
Major loss of function	60929 (17.5%)	8282 (30.8%)	69211 (18.4%)			83236 (13.6%)	9456 (23%)	92692 (14.2%)		
Extreme loss of function	15335 (4.4%)	2607 (9.7%)	17942 (4.8%)			17331 (2.8%)	2475 (6%)	19806 (3%)		
Hospital bed size				<0.001					<0.001	
Small	43352 (12.4%)	3940 (14.7%)	47292 (12.6%)			79894 (13.1%)	5371 (13.1%)	85265 (13.1%)		
Medium	67499 (19.4%)	4281 (15.9%)	71780 (19.1%)			155932 (25.5%)	8575 (20.9%)	164507 (25.2%)		
Large	237820 (68.2%)	18645 (69.4%)	256465 (68.3%)			376363 (61.5%)	27083 (66%)	403446 (61.8%)		
Control/Ownership of Hospital				<0.001					<0.001	
Government, nonfederal	45552 (13.1%)	3480 (13%)	49032 (13.1%)			75764 (12.4%)	5392 (13.1%)	81156 (12.4%)		
Private, not-profit	281408 (80.7%)	22469 (83.6%)	303877 (80.9%)			475194 (77.6%)	32402 (79%)	507596 (77.7%)		
Private, invest-own	21710 (6.2%)	917 (3.4%)	22627 (6%)			61231 (10%)	3235 (7.9%)	64466 (9.9%)		
Hospital Urban Rural Designation				<0.001					<0.001	
Large metropolitan areas with at least 1 million residents	221459 (63.5%)	17510 (65.2%)	238969 (63.6%)			352060 (57.5%)	25157 (61.3%)	377217 (57.7%)		
Small metropolitan areas with less than 1 million residents	116184 (33.3%)	9051 (33.7%)	125235 (33.3%)			222897 (36.4%)	14524 (35.4%)	237421 (36.3%)		
Micropolitan areas	8449 (2.4%)	266 (1%)	8715 (2.3%)			29788 (4.9%)	1101 (2.7%)	30889 (4.7%)		
Not metropolitan or micropolitan (non-urban residual)	2579 (0.7%)	40 (0.1%)	2619 (0.7%)			7444 (1.2%)	247 (0.6%)	7691 (1.2%)		
Hospital teaching status				<0.001					<0.001	
Metropolitan non-teaching hospital	25368 (7.3%)	1403 (5.2%)	26771 (7.1%)			81564 (13.3%)	3986 (9.7%)	85550 (13.1%)		
Metropolitan teaching hospital	312275 (89.6%)	25157 (93.6%)	337432 (89.9%)			493393 (80.6%)	35695 (87%)	529088 (81%)		
Non-metropolitan hospital	11027 (3.2%)	306 (1.1%)	11333 (3%)			37232 (6.1%)	1349 (3.3%)	38581 (5.9%)		
Discharge destination				<0.001					<0.001	
Home/self-care	330015 (94.7%)	24337 (90.6%)	354352 (94.4%)			570630 (93.2%)	37342 (91%)	607972 (93.1%)		
Home health care	2892 (0.8%)	398 (1.5%)	3290 (0.9%)			4568 (0.7%)	526 (1.3%)	5094 (0.8%)		
Discharge against medical advice	308 (0.1%)	10 (0%)	318 (0.1%)			3067 (0.5%)	373 (0.9%)	3440 (0.5%)		
Length of stay and cost analysis										
Index admission length of stay(days) (median[IQR])	2(1-4)	4(2-6)	3(1-5)			3(2-5)	4(2-6)	3(2-5)		
Index admission hospital charge (U.S.$) (median[IQR])	20467(10980-41052)	27228(14197-56512)	20822(11166-41975)			18676(10552-37076)	23152(12411-46656)	18907(10640-37660)		
Index admission cost (U.S.$) (median[IQR])	5763(3146-11706)	8052(4119-17069)	5896(3199-12021)			5113(3054-10021)	6353(3436-13233)	5177(3070-10231)		
Re-admission length of stay(days) (median[IQR])			2 [2 – 6]					2 [2 – 6]		
Re-admission hospital charge (U.S.$) (median[IQR])			11453 [13392 – 45387]					21777 [12124 – 41613]		
Re-admission cost (U.S.$) (median[IQR])			7230 [3933 – 13920]					5987 [3388 – 11756]		

Table 3 presents the most frequent diagnosis associated with readmissions for all age groups.

**Table 3:** Causes and frequencies of primary diagnosis category for readmissions encounters

Age <1		Age 1-4		Age 5-12		Age 13-18	
Diagnosis category	Frequency (%) n=76,274	Diagnosis category	Frequency (%) n=21,340	Diagnosis category	Frequency (%) n=26,723	Diagnosis category	Frequency (%) n=41,180
Hemolytic jaundice	20338 (26.7%)	Conditions due to neoplasm or the treatment of neoplasm/antineoplastic therapies	3157 (14.8%)	Conditions due to neoplasm or the treatment of neoplasm antineoplastic therapies	3995 (15%)	Depressive disorders	4696 (11.4%)
Other unspecified perinatal conditions	9140 (12%)	Diseases of white blood cells	1390 (6.5%)	Complication of other surgical or medical care, injury, initial encounter	1533 (5.7%)	Conditions due to neoplasm or the treatment of neoplasm / antineoplastic therapies	4083 (9.9%)
Perinatal infections	6960 (9.1%)	Complication of other surgical or medical care, injury, initial encounter	1176 (5.5%)	Diseases of white blood cells	1447 (5.4%)	Sickle cell trait/anemia	2303 (5.6%)
Acute bronchiolitis	5236 (6.9%)	Epilepsy; convulsions	1022 (4.8%)	Other specified and unspecified mood disorders	1019 (3.8%)	Bipolar and related disorders	1753 (4.3%)
Neonatal digestive and feeding disorders	3223 (4.2%)	Pneumonia (except that caused by tuberculosis)	945 (4.4%)	Sickle cell trait/anemia	1008 (3.8%)	Diabetes mellitus with complication	1717 (4.2%)
Respiratory perinatal condition	2614 (3.4%)	Respiratory failure; insufficiency; arrest	932 (4.4%)	Epilepsy; convulsions	884 (3.3%)	Complication of other surgical or medical care, injury, initial encounter	1428 (3.5%)
Digestive congenital anomalies	2075 (2.7%)	Acute bronchiolitis	859 (4%)	Depressive disorders	706 (2.6%)	Other specified and unspecified mood disorders	1379 (3.3%)
Other general signs and symptoms	1989 (2.6%)	Asthma	852 (4%)	Pneumonia (except that caused by tuberculosis)	695 (2.6%)	Diseases of white blood cells	942 (2.3%)
Respiratory failure; insufficiency; arrest	1709 (2.2%)	Other specified upper respiratory infections	758 (3.6%)	Asthma	617 (2.3%)	Schizophrenia spectrum and other psychotic disorders	849 (2.1%)
Other specified upper respiratory infections	1258 (1.6%)	Disorders of fluid and electrolytes	745 (3.5%)	Intestinal infections	533 (2%)	Complications during the puerperium	772 (1.9%)

Predictors of 30-day readmission

Table 4 presents the results of logistic regression analysis for the predictors of 30-day readmission for all age groups.

**Table 4:** Logistic regression analysis for 30-day readmissions Abbreviations: UTI: Urinary Tract Infection; IBD: Inflammatory Bowel Disease; APR DRG: All Patient Refined-Diagnosis Related Group

	Age <1		Age 1-4		Age 5-12		Age 13-18	
	P value	Odds ratio	P value	Odds ratio	P value	Odds ratio	P value	Odds ratio
Primary expected payer (Reference: Medicaid)								
Medicare	<0.001	0.588 (0.512 - 0.674)	<0.001	2.166 (1.826 - 2.569)	<0.001	2.127 (1.834 - 2.467)	<0.001	1.364 (1.188 - 1.566)
Private	<0.001	0.790 (0.778 - 0.802)	0.289	1.016 (0.986 - 1.047)	0.049	1.027 (1.000 - 1.054)	0.005	0.980 (0.960 - 1.001)
Self-pay	<0.001	0.635 (0.607 - 0.664)	<0.001	0.678 (0.602 - 0.763)	0.575	0.973 (0.884 - 1.071)	<0.001	0.775 (0.720 - 0.834)
No charge	<0.001	0.475 (0.325 - 0.694)	0.994	1.002 (0.546 - 1.839)	0.340	0.809 (0.523 - 1.251)	0.216	0.808 (0.577 - 1.131)
Other	<0.001	0.811 (0.774 - 0.850)	0.001	0.850 (0.774 - 0.933)	0.550	1.023 (0.950 - 1.101)	0.300	0.971 (0.916 - 1.029)
Quartile of median household income (Reference: 0-25^th^ centile)								
26^th^ –50^th^	0.001	1.034 (1.013 - 1.055)	<0.001	1.110 (1.071 - 1.150)	0.379	0.986 (0.954 - 1.018)	0.003	1.029 (1.003 - 1.056)
51^st^–75^th^	<0.001	1.045 (1.024 - 1.066)	<0.001	1.107 (1.066 - 1.150)	0.444	0.987 (0.954 - 1.021)	0.001	1.036 (1.008 - 1.065)
76^th^ –100^th^	0.148	0.984 (0.963 - 1.006)	<0.001	0.911 (0.871 - 0.953)	0.054	1.037 (0.999 - 1.077)	0.001	1.037 (1.006 - 1.069)
Comorbidities (Reference: no)								
Dehydration	<0.001	0.794 (0.755 - 0.836)	<0.001	0.636 (0.603 - 0.670)	<0.001	0.807 (0.767 - 0.848)	<0.001	0.861 (0.823 - 0.900)
Cancer	<0.001	1.745 (1.657 - 1.836)	<0.001	6.217 (5.989 - 6.454)	<0.001	5.453 (5.278 - 5.634)	<0.001	4.892 (4.745 - 5.043)
Injury/Poisoning	<0.001	0.382 (0.279 - 0.522)	<0.001	0.476 (0.352 - 0.644)	<0.001	0.323 (0.248 - 0.421)	<0.001	0.589 (0.523 - 0.663)
Transplant		2.061 (1.502 - 2.830)	<0.001	1.942 (1.789 - 2.108)	<0.001	1.704 (1.587 - 1.829)	<0.001	1.400 (1.284 - 1.526)
UTI	<0.001	1.307 (1.212 - 1.408)	<0.001	1.026 (0.949 - 1.110)	<0.001	1.190 (1.115 - 1.270)	0.523	0.983 (0.932 - 1.037)
Renal disorders	<0.001	1.697 (1.563 - 1.843	<0.001	1.553 (1.437 - 1.677)	<0.001	1.668 (1.572 - 1.770)	<0.001	1.451 (1.381 - 1.525)
Sickle cell disease	<0.001	2.625 (2.369 - 2.909)	<0.001	1.678 (1.530 - 1.841)	<0.001	2.413 (2.275 - 2.560)	<0.001	3.890 (3.721 - 4.066)
Cardiomyopathy and dysrrhythmias	<0.001	1.339 (1.237 - 1.449)	<0.001	1.367 (1.234 - 1.515)	0.007	1.149 (1.038 - 1.272)	<0.001	1.219 (1.136 - 1.309)
Intestinal infection	<0.001	1.416 (1.295 - 1.549)	0.081	0.941 (0.878 - 1.008)	0.013	1.093 (1.019 - 1.172)	0.005	1.117 (1.035 - 1.206)
Appendiceal conditions	-	-	<0.001	1.332 (1.152 - 1.541)	0.101	1.056 (0.990 - 1.126)	0.277	0.964 (0.901 - 1.030)
IBD	-	-	0.903	0.973 (0.623 - 1.518)	<0.001	2.131 (1.920 - 2.366)	<0.001	1.893 (1.778 - 2.017)
Hepatitis	-	-	<0.001	1.770 (1.096 - 2.859)	<0.001	1.702 (1.286 - 2.254)	<0.001	1.779 (1.483 - 2.135)
Digestive and heptobiliary diseases	<0.001	2.264 (2.199 - 2.330)	<0.001	1.658 (1.606 - 1.712)	<0.001	1.417 (1.377 - 1.458)	<0.001	1.427 (1.393 - 1.463)
Epilepsy	<0.001	2.707 (2.556 - 2.866)	<0.001	1.622 (1.557 - 1.690)	<0.001	1.439 (1.384 - 1.497)	<0.001	1.339 (1.287 - 1.394)
Diabetes	0.064	0.472 (0.214 - 1.044)	<0.001	0.717 (0.582 - 0.884)	<0.001	0.841 (0.776 - 0.910)	<0.001	1.468 (1.407 - 1.531)
Endocrine causes other than diabetes	<0.001	1.948 (1.878 - 2.020)	<0.001	1.360 (1.308 - 1.415)	<0.001	1.383 (1.339 - 1.429)	<0.001	1.246 (1.214 - 1.278)
Cystic fibrosis	0.001	0.117 (0.056 - 0.246)	0.024	0.721 (0.543 - 0.958)	<0.001	0.675 (0.566 - 0.804)	<0.001	0.832 (0.745 - 0.928)
Skin and Subcutaneous	<0.001	1.342 (1.286 - 1.400)	<0.001	0.898 (0.859 - 0.939)	<0.001	0.929 (0.893 - 0.967)	<0.001	0.961 (0.928 - 0.995)
Psychiatric disorders	<0.001	1.320 (1.063 - 1.640)	0.705	0.975 (0.855 - 1.112)	<0.001	1.634 (1.580 - 1.689)	<0.001	1.272 (1.244 - 1.300)
Congenital anomalies	<0.001	1.449 (1.423 - 1.475)	<0.001	1.554 (1.501 - 1.610)	<0.001	1.108 (1.067 - 1.152)	<0.001	1.135 (1.090 - 1.182)
Musculoskeletal	0.577	0.962 (0.839 - 1.103)	<0.001	0.736 (0.661 - 0.819)	<0.001	0.777 (0.728 - 0.828)	<0.001	0.779 (0.741 - 0.819)
Complications during childbirth		-	-	-	-	-	<0.001	0.294 (0.275 - 0.313)
Asthma and bronchiolitis	<0.001	1.731 (1.667 - 1.798)	<0.001	0.870 (0.837 - 0.904)	<0.001	0.881 (0.852 - 0.911)	0.078	1.026 (0.997 - 1.056)
Pneumonia	0.007	1.170 (1.102 - 1.241)	<0.001	0.790 (0.750 - 0.832)	<0.001	0.773 (0.730 - 0.819)	0.520	0.979 (0.919 - 1.044)
Respiratory failure and arrest	<0.001	1.476 (1.406 - 1.550)	<0.001	1.398 (1.331 - 1.468)	<0.001	1.235 (1.163 - 1.310)	0.003	1.101 (1.033 - 1.173)
Hemolytic and perinatal jaundice	<0.001	1.306 (1.285 - 1.328)	-	-	-	-	-	-
Septicemia	<0.001	0.802 (0.731 - 0.880)	0.295	0.953 (0.872 - 1.043)	<0.001	0.936 (0.862 - 1.017)	0.002	0.896 (0.835 - 0.961)
Neutropenia	<0.001	1.516 (1.393 - 1.650)	0.830	1.008 (0.940 - 1.080)	<0.001	0.982 (0.918 - 1.050)	0.218	1.052 (0.971 - 1.139)
APR DRG Mortality risk (Reference: minor)								
Moderate likelihood of dying	<0.001	3.069 (2.984 - 3.157)	<0.020	3.417 (3.312 - 3.526)	<0.001	3.454 (3.360 - 3.552)	<0.001	3.010 (2.935 - 3.086)
Major likelihood of dying	<0.001	4.719 (4.559 - 4.884)	<0.001	4.013 (3.831 - 4.203)	<0.001	3.177 (3.031 - 3.330)	<0.001	2.711 (2.596 - 2.832)
Extreme likelihood of dying	<0.001	5.574 (5.250 - 5.919)	<0.001	2.694 (2.459 - 2.951)	<0.001	2.505 (2.288 - 2.742)	<0.001	1.897 (1.748 - 2.060)
APR DRG Severity of Illness (Reference: minor)								
Moderate loss of function	<0.001	1.472 (1.447 - 1.498)	<0.001	2.668 (2.560 - 2.780)	<0.001	2.308 (2.230 - 2.389)	<0.001	1.846 (1.800 - 1.894)
Major loss of function	<0.001	2.228 (2.180 - 2.276)	<0.001	5.311 (5.095 - 5.536)	<0.001	3.797 (3.662 - 3.938)	<0.001	2.888 (2.804 - 2.975)
Extreme loss of function	<0.001	6.272 (6.078 - 6.473)	<0.001	6.755 (6.403 - 7.126)	<0.001	4.749 (4.517 - 4.994)	<0.001	3.631 (3.465 - 3.805)
Hospital bed size (Reference: small)								
Medium	<0.001	0.879 (0.858 - 0.900)	<0.001	0.603 (0.572 - 0.635)	<0.001	0.698 (0.667 - 0.730)	<0.001	0.818 (0.790 - 0.847)
Large	<0.001	1.201 (1.176 - 1.226)	<0.001	0.850 (0.818 - 0.884)	<0.001	0.863 (0.832 - 0.894)	<0.001	1.070 (1.038 - 1.103)
Control/Ownership of Hospital (Reference: government, nonfederal)								
Private, not-profit	<0.001	0.743 (0.726 - 0.760)	0.001	1.077 (1.031 - 1.124)	0.020	1.045 (1.007 - 1.085)	0.001	0.958 (0.930 - 0.987)
Private, invest-own	<0.001	0.532 (0.513 - 0.553)	<0.001	0.456 (0.415 - 0.502)	<0.001	0.553 (0.513 - 0.596)	<0.001	0.742 (0.710 - 0.776)
Hospital Urban Rural Designation (Reference:Large metropolitan areas with at least 1 million residents)								
Small metropolitan areas with less than 1 million residents	<0.001	0.901 (0.888 - 0.915)	<0.001	0.814 (0.789 - 0.839)	0.269	0.985 (0.960 - 1.012)	<0.001	0.912 (0.893 - 0.931)
Micropolitan areas	<0.001	0.600 (0.579 - 0.621)	<0.001	0.320 (0.281 - 0.365)	<0.001	0.398 (0.352 - 0.450)	<0.001	0.517 (0.487 - 0.550)
Not metropolitan or micropolitan (non-urban residual)	<0.001	0.525 (0.490 - 0.562)	<0.001	0.238 (0.186 - 0.303)	<0.001	0.195 (0.143 - 0.267)	<0.001	0.465 (0.410 - 0.528)
Hospital teaching status (Reference: Non-metropolitan hospital)								
Metropolitan non-teaching hospital	<0.001	1.451 (1.402 - 1.502)	<0.001	1.822 (1.596 - 2.079)	<0.001	1.994 (1.759 - 2.262)	<0.001	1.349 (1.267 - 1.436)
Metropolitan teaching hospital	<0.001	1.699 (1.646 - 1.753)	<0.001	3.254 (2.900 - 3.651)	<0.001	2.905 (2.591 - 3.257)	<0.001	1.997 (1.889 - 2.111)

For children less than one year old, the overall diagnoses for pediatric readmission in this age group, excluding live birth, were epilepsy/seizures [2.707 (2.556 - 2.866), p<0.001], gastrointestinal ailments including reflux/milk protein allergy [2.264 (2.199 - 2.330), p<0.001] and sickle cell disease [2.625 (2.369 - 2.909), p<0.001]. It was also seen that dehydration, septicemia, cystic fibrosis and injury/poisonings were negative predictors for readmission in this age group.

**Figure 1:**
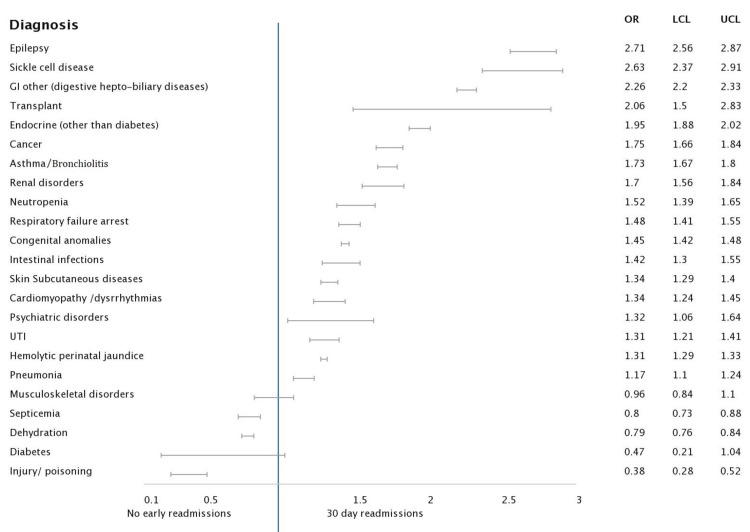
Forest plot analysis of pertinent diagnosis associated with 30-day readmission: Age group <1 year Abbreviations: GI: gastrointestinal; UTI: urinary tract infection

**Figure 2:**
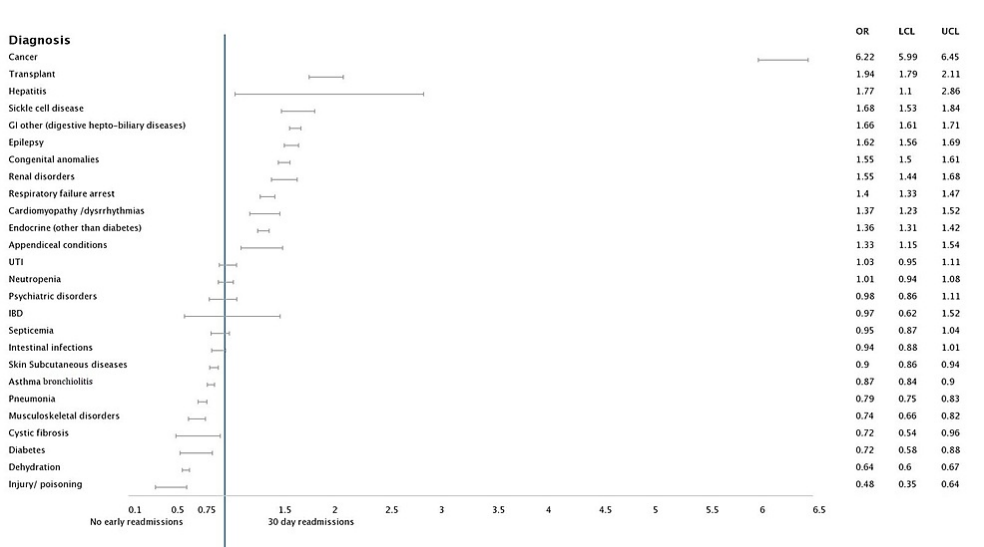
Forest plot analysis of pertinent diagnosis associated with 30-day readmission: Age group 1-4 years Abbreviations: GI: Gastrointestinal; UTI: urinary tract infection; IBD: Inflammatory bowel disease

**Figure 3:**
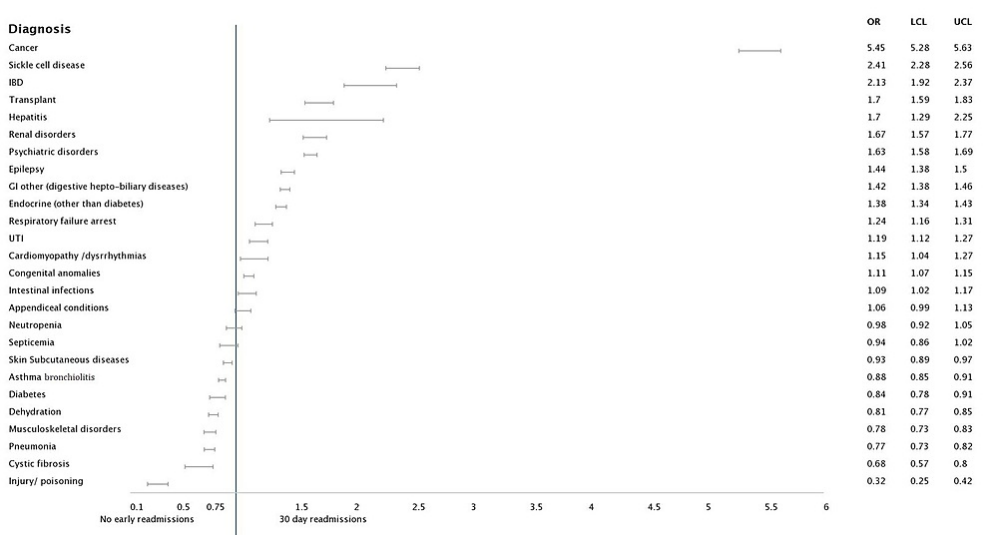
Forest plot analysis of pertinent diagnosis associated with 30-day readmission: Age group 5-12 years Abbreviations: IBD: Inflammatory bowel disease; UTI: Urinary tract infection

**Figure 4:**
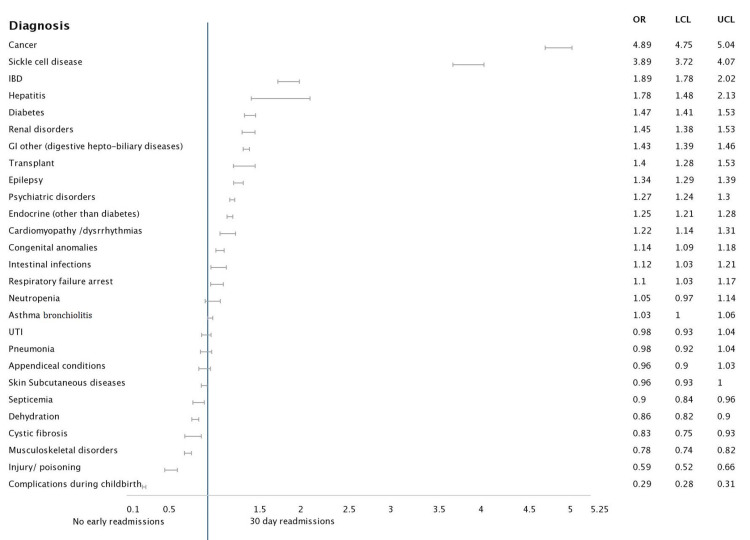
Forest plot analysis of pertinent diagnosis associated with 30-day readmission: Age group 13 years and above Abbreviations: IBD: Inflammatory bowel disease; GI: Gastrointestinal; UTI: Urinary tract infection

Under the next category including ages one to four years old, the predominant causes for comorbidities associated with admission were cancer [6.217 (5.989 - 6.454), p<0.001], transplant recipients [1.942 (1.789 - 2.108), p<0.001], hepatitis [1.770 (1.096 - 2.859), p<0.001]. Children admitted with causes such as asthma, bronchiolitis, pneumonia, diabetes and dehydration showed lesser chances of readmission (Figures 1, 2, 3, 4).

In the third category of ages between five and 12 years, the highest odds of occurrence of readmission were with comorbidities namely cancer [5.453 (5.278 - 5.634, p<0.001], sickle cell diseases [2.413 (2.275 - 2.560), p<0.001] and inflammatory bowel diseases (IBD) [2.131 (1.920 - 2.366), p<0.001]. There was a multitude of admission comorbidities with odds against readmission for this age group, top causes of which included pneumonia, cystic fibrosis and musculoskeletal injuries.

For adolescent ages, the highest associations with readmission were under cancer [4.892 (4.745 - 5.043), p<0.001], sickle cell disease [3.890 (3.721 - 4.066), p<0.001], renal diseases [1.451 (1.381 - 1.525), p<0.001] and IBD [1.893 (1.778 - 2.017), p<0.001]. Another important association was psychiatric illnesses [1.272 (1.244 - 1.300), p<0.001] which had not been seen in the previous groups. The patients who showed a negative prediction for readmission were composed of groups with co-morbidities namely cystic fibrosis, musculoskeletal causes, skin diseases, dehydration and injuries/poisonings.

## Discussion

In this nationwide represented database for the pediatric population, we have the opportunity to analyze a large sample size which gives us a more reliable outcome. To the best of our knowledge this is a first pediatric, age-defined (including infants), all-cause, all-payer review of national data from the Healthcare Utilization Project, Nationwide Readmission Database using around 5.5 million inpatient encounters with ICD-10 data.

Using nationally representative data for U.S. rehospitalizations, the biggest trend we demonstrated through this study was increasing rates of unplanned readmissions with cancer as a comorbidity across all age groups. This corroborates with previous studies which have time and again shown cancer and transplant recipients to be a higher association for readmissions [15]. The total rate of readmission is 3.2% for all ages inclusive of ages less than one. For those older than one year of age, the rate of readmission is 6.7%. In comparison to previous years (2010-2016) this number has evolved from 6.26 to 7.02% to 6.7% in our study, hence there is some improvement in health care interventions and strategies on the national scale as seen by the improvement in rates of readmission [16]. Another independent study conducting readmission rate assessment for 72 free-standing pediatric hospitals in 2013 showed a rate of 6.2% which might be attributed to the fact that the hospitals may not have had sicker patients at the time [17].

While the causes are individually different under different age categories, there were a few patterns noted in our study. While chronic complex ailments such as sickle cell diseases and cancer have significant associated comorbidities which continue to show an increasing rate for ages above one. Cohen et al. and Sehgal et al. had also demonstrated similar trends through their studies [18,19]. Besides the illness severity which may be a valid cause, there is a continuous need for improvement in patient compliance and education for these groups of children which might help in improving readmission rates [20]. The prevalence of children with chronic conditions is increasing because of improved survival in the neonatal period and medical advances in care and technology ultimately leading to additional medical needs and inpatient stays in future [21]. By making comprehensive discharge protocols including provision of discharge medications in hand at the time of hospital discharge, dedicated education at the time of discharge and close follow-up, unplanned readmissions can be potentially reduced [22].

Bardach et al. reported in 2013 that the pediatric condition-specific readmission rates across various hospitals may not be a useful measure for comparison of performances since certain institutional places will always have a higher rate due to it being a bigger center for admission for especially chronic medical ailments [23]. They also suggested that few hospitals that care for children are identified more or less popular for revisits, even for common pediatric diagnoses, likely due to low hospital volumes. Bucholz et al. previously described trends for readmission rates from 2010-2016 [15]. They determined that, although the total number of hospital admissions declined over these years, however the readmission rates were found to have increased. They also found that the higher readmission rates were associated with chronic diseases, which has been reiterated through our study with most contemporary data. Recently they also identified the impact of varying patient insurances over the rates of readmission. It was seen that the readmission rates for Medicaid- and privately insured pediatric patients declined slightly from 2010 to 2017 but continued to remain higher relatively and reiterated in our study amongst the Medicaid beneficiaries indicating the role of insurance affecting the services and need for rehospitalization [24].

It has been noted that in ages one to four years dehydration along with asthma or bronchiolitis, which are one of the most common diagnoses in the pediatric population across the United States, are negative predictors of unplanned readmission. This might be possible due to adequate close follow-up and use of preventive measures like Asthma Action Plans, good asthma scoring by more and more institutions can lead to reduction in readmissions [25]. Asthma by itself has however been shown to have higher rates of readmission but that may just be reflecting the negative predictor for this age group alone as illustrated from our study [26]. Psychiatric illnesses including anxiety disorders, depression and suicidal ideation tend to come up as one of the forthcoming causes in the older age groups which leads to higher rehospitalizations. This is another important focus for all future approaches and plans of action. Sehgal et al. recently demonstrated urinary tract infections to be negative predictors of readmission for this age group which might also be attributed to close outpatient follow-up which has been illustrated with our study [19]. Richards et al. demonstrated the importance of the internet as a means of managing depressive symptoms through the CATCH-IT trial. They reported through their analysis that greater motivation for depression prevention and lower ratings of self-efficacy at baseline were associated with greater declines in depression symptoms [27]. Inflammatory bowel diseases are also a significant burden for this age group and amongst patients of Crohn’s disease, psychiatric causes are a frequent cause for readmission [28].

Among infants, factors like hyperbilirubinemia and bronchiolitis are associated with increased unplanned readmissions. It may be interesting to consider sub-threshold phototherapy for neonates during birth hospitalizations as a measure that has been previously studied, which might contribute to its overuse [29]. Readmission rates of bronchiolitis in previous studies have ranged from 1-4%, which when compared to overall pediatric readmissions appears to be on the lower spectrum [30]. The higher rates were also seen for reflux, milk protein allergy and other gastrointestinal disorders in this group which is an important causal association and has been less studied due to lesser research for this age group. 

There are certain factors that will always continue to affect outcomes but may not be accounted for such as parental perception. Multiple times it might be considered that a child is fit for discharge but a parent may feel that they are not healthy enough which is an important consideration for the pediatric population [31]. On the other hand there are certain factors that may seem to influence readmission but they actually do not largely affect it. Krumholz et al. also determined from their study that the rate of readmission is not related to increased mortality rates, by which we can safely say that early triaging and rapid management and interventions may not play a role in readmission rate [32].

Moving forward, we need further investigations to understand the reasons for variation in readmission rates across children’s hospitals. There may be subtle variations in the form of care during the index hospitalization as well as post-discharge care which could be a contributing factor. The bigger hospitals catering to a larger population have historically had higher rates of unplanned readmissions likely because they are more widely available and accessible [33]. Multiple variables at the patient, family and community level may influence readmission risk and thus require investigation. Use of artificial intelligence should be employed for predicting readmission rates for better validation and accuracy as demonstrated by Amritphale et al in their recent study [34]. This is one of the leading algorithms for research and is causing scientists to rethink how we integrate information and analyze data. 

Furthermore, work is needed to determine how to measure the most relevant variables accurately and reliably using available data sources. Factors such as access to primary care for follow-up visits or community factors such as parental ability to avail paid leaves may also play a role. In addition, there can be unpredictable disease progression which may govern increased length of stay and may affect readmission rates as well [35].

NRD is in a format of annualized data where each patient could have a maximum follow-up duration of only one year and the same patient cannot be followed over the years even if data from multiple years are combined. By design, NRD does not allow determination of regional variations within the dataset. This study does not give a glimpse into patients with various causes as an outpatient procedure and represents only hospital admissions. As with any observational data, the results do not suggest a causal relationship as there can be other unmeasured confounders. NRD data does not include the ‘observation stays’ in our analysis that might alter the total number of readmissions but would be unlikely to change the overall trend in readmission rates after inpatient admissions. Lastly, the NRD database does not provide pharmacological data that may impact readmissions. Also being an administrative database there is always a possibility that the ICD-10 codes may or may not be completely accurate.

## Conclusions

Our analysis shows the overall readmission rates are 3.2% but for those older than one year of age, the rates were higher at 6.7%. The chronic complex medical conditions, cancer and sickle cell disease account for greater readmission rates across all age groups along with psychiatric disorders and inflammatory bowel diseases being an important association for older age groups. This study will pave the way for more comprehensive strategies to help identify patients at high risk of unplanned readmission for targeted interventions. This will also help control and increase savings to the wider health care economy with total charges for unplanned readmissions amounting to over $70 billion.
